# Comparative Assessment of the Pharmaceutical Quality of Amoxicillin Capsules Marketed in Authorized and an Unregulated Point of Sale in Mexican Market

**DOI:** 10.1007/s43441-026-00978-0

**Published:** 2026-04-28

**Authors:** Gael Marmolejo-Bernal, Leny Mejía-Amaro, Teresa Benítez-Escamilla, Alberta Lourdes Castillo-Granada, Leticia Cruz-Antonio

**Affiliations:** Facultad de Estudios Superiores Zaragoza, UNAM, Av. Guelatao No. 66, Colonia Ejército de Oriente, Iztapalapa, 09230 Ciudad de México, Mexico

**Keywords:** Pharmaceutical quality, Amoxicillin, Mexican market, Consumer safety, Unregulated markets, Substandard product

## Abstract

**Background:**

Purchasing medicines outside of points of sale regulated by competent authorities increases the likelihood of exposure to substandard or falsified products. Amoxicillin, a widely used essential antibiotic, has been particularly associated with this issue. However, evidence from middle-income countries such as Mexico remains limited. This study evaluated the pharmaceutical quality of 500 mg amoxicillin capsules purchased from an unregulated point of sale in the Mexican market by assessing compliance with official quality tests, including dissolution profiles.

**Methodology:**

An exploratory comparative study was used, considering two fixed factors: point of sale (regulated or unregulated) and brand. Seven groups were evaluated, representing combinations of these factors, except for one brand unavailable in the regulated market. Samples were subjected to pharmacopoeial quality control tests and in vitro dissolution studies. Due to limited sample availability from unregulated sources, some pharmacopoeial sequential procedures could not be fully completed. Dissolution profiles were compared using similarity factor (f2), dissolution efficiency, and mean dissolution time.

**Results:**

Medicines from the unregulated point of sale showed greater variability in quality attributes than those from regulated establishments. Some products exhibited deviations in assay, elevated L1 value in dose unit uniformity, and differences in dissolution performance. One sample showed suggestive evidence of an expiration date tampering. Significant differences in dissolution profiles were observed between certain formulations and the reference product.

**Conclusion:**

Although full pharmacopoeial procedures were not completed in all cases, the findings indicated variability and potential inconsistencies in quality, highlighting the need to strengthen regulatory oversight and further studies.

## Introduction

Ensuring the availability of safe, effective, and high-quality medicines for all remains an unfulfilled global goal, despite numerous implemented policies. For this reason, the World Health Organization (WHO) and national health regulatory authorities have emphasized this issue. Unfortunately, not all products marketed as medicines meet the required quality attributes, posing a widespread and alarming public health problem [1].

According to the WHO, the health risks and therapeutic failures caused by medicines that do not meet quality standards, including substandard medical products, defined as medicines that do not meet quality standards and specifications, often due to poor manufacturing practices or inadequate quality control, and, in more severe cases, falsified medical products, which deliberately misrepresent their identity, composition, or source, are most evident in low- and middle-income countries [2] both definitions are consistent with Mexican health legislation.

Mexico as part of this concerning scenario, has been identified since 2018 as one of the six countries with the highest sales of substandard medical products (SMP) and falsified medical products (FMP) worldwide [3]. The sale and distribution of SMP and FMP typically occur through unregulated or informal points of sale, including physical or online marketplaces, where a wide range of medicines is offered, from analgesics to antibiotics [4, 5]. Among these, the Tepito Market represents one of the largest and most accessible unregulated markets in Mexico City, despite the existence of local regulations, there is a lack of sanitary and administrative controls, operating outside the formal authorizations required for fixed establishments, and characterized by a high demand for medicines sold outside regulatory oversight [6]. For this reason, and given its accessibility and documented consumer demand, this Tepito market was selected as a representative unregulated point of sale for the present comparative exploratory evaluation.

Amoxicillin is a first-line antibiotic used in both adults and children to treat 10 of the 12 most common conditions managed in primary care across all WHO regions. However, this antibiotic is also subject to the informal marketing of SMP and FMP in both physical and online settings [7]. International reports estimate that approximately 17% of amoxicillin and other antibiotic formulations marketed globally are of poor quality or falsified [8]. This situation not only leads to frequent health alerts [9] but also represents a significant risk of therapeutic failure. In the case of amoxicillin, subtherapeutic dosing contributes to the development of antimicrobial resistance and causes economic losses to patients due to ineffective treatments [10].

Several studies have reported failures in medicines purchased outside regulated distribution channels, mainly in African and Latin American countries, where health surveillance is often limited or reactive [11]. However, direct evidence linking medicinal products quality to the place of purchase remains limited, especially regarding amoxicillin in the Mexican market.

This exploratory and comparative study aimed to compare the pharmaceutical quality of 500 mg amoxicillin capsules purchased from authorized pharmacies with those bought from an unregulated point of sale in Mexico City specifically, an unregulated market known as Tepito tianguis. The study provides evidence of noncompliance with pharmaceutical quality standards among amoxicillin capsules sold in unregulated markets in Mexico, highlighting the potential health risks associated with insufficient regulatory control and limited health surveillance.

## Materials and Methods

### Reagents

Chromatographic-grade acetonitrile and the amoxicillin reference substance were purchased (99% anhydrous basis) from Sigma-Aldrich (Germany). Analytical grade reagents: Potassium phosphate monobasic and Sodium hydroxide were purchased from (Meyer Chemistry Reagents, Mexico City). Water was obtained from a Milli-Q Synthesis water purification system (Millipore, Billerica, MA, USA).

### Sample Collection

Samples of amoxicillin capsules of 500 mg were collected in Mexico City between January and April 2024. Four different commercial brands were purchased from an unregulated physical market (Tepito tianguis). This site was selected as a representative unregulated point of sale where prescription medicines are routinely offered without authorisation or prescription [6]. Additionality three of the four brands were purchased from retail pharmacies that complied with health regulations. The brands were coded as AN, BN, CN, and DN (unregulated market) and AA, BA, and DA (authorized pharmacies), respectively. Among the formally obtained samples, brand DA corresponded to the reference product. Detailed information about the samples is presented in Table 1. All medical products obtained for this study, whether purchased from authorized pharmacies or unregulated market, were manufactured in Mexico.

**Table 1:** 500 mg amoxicillin capsule samples included in the study

Code	Batch number	Source of purchase	Expiration date	Health registration (declared on packaging)*	Verified regulatory status**
AN	Q0523391	Unregulated market	May 2025	Yes	Valid
BN	C3326	Unregulated market	Jun 2025	Yes	Valid
CN	45,102,008	Unregulated market	Oct 2026	Yes	Cancelled (non-valid registration)
DN	043AA010VA	Unregulated market	Dec 2024	Yes	Valid
AA	Q0623530	Authorized pharmacy	Jun 2025	Yes	Valid
BA	C4078	Authorized pharmacy	Feb 2026	Yes	Valid
DA***	043KZ08	Authorized pharmacy	Jun 2025	Yes	Valid

^*^Refers to the information printed on the product label. ** Status verified via the official COFEPRIS database (Federal Commission for Protection against Sanitary Risks). A “cancelled” status indicates no valid marketing authorisation was held at the time of analysis. ***Reference product according to the COFEPRIS National Reference Medicines List

### Quality Control. Physicochemical Evaluations

#### External Aspect

A visual inspection test was performed as a quality control procedure. Ten units of each capsule brand were placed in a Petri dish and examined under uniform white light against a colored background. The capsules were evaluated for external characteristics, including color, shape, surface defects, and physical integrity [12, 13].

#### Identity Test

The retention time obtained from the chromatogram of each capsule sample was compared with that of reference preparation, following the procedure established in the pharmacopoeial assay for active content determination [14].

#### Dose Uniformity

Ten capsules of each brand were individually weighed on an analytical balance (Bel Engineering M5-m2145ai-ion, Monza, Italy). Each capsule was carefully opened without losing any part of the shell, and its contents were completely emptied. The empty capsules were then weighed, and the net weight was calculated by subtracting the shell weight from the gross capsule weight. The active ingredient content in each capsule was determined using the net weight and assay test results. The results were expressed as a percentage of the declared dose and compared with the acceptance value (L1). An L1 value of ≤ 15 was considered acceptable.[14].

#### Disintegration Test

The disintegration test was performed using a BJ2 disintegrator (Guoming Medicinal Equipment Co., Ltd., Tianjin, China). Six capsules of each brand were immersed in 900 mL of distilled water maintained at 37 ± 2 °C. The time at which each capsule completely disintegrated was recorded [14].

#### Assay

The amoxicillin content in the capsules of each brand was determined according to the Mexican Pharmacopoeia monograph, with minor modifications [14]. To improve efficiency and resolution, the original column was replaced by a shorter one (150 mm) and by a smaller particle size (3.5 µm). Due to limited sample availability, preparation was carried out using a 10 capsules pool representation, instead of the 20 units suggested in the pharmacopoeial method [14].

*Diluent solution*. Monobasic potassium phosphate solution (13.6 g of salt in 2 L of water) adjusted to pH 5.0 ± 0.1 with sodium hydroxide solution.

*Reference Solution*. A reference standard solution of amoxicillin trihydrate was prepared in the diluent to a concentration of 0.1 mg/mL.

*Test solution*. The contents of ten pre-weighed capsules were mixed. A portion equivalent to 25 mg of amoxicillin was transferred to a 25 mL volumetric flask, dissolved, and diluted to volume with the diluent solution using sonication for 10 min. Appropriate dilutions were subsequently prepared to obtain a final concentration of 0.1 mg/mL. Both reference and test solutions were filtered through a 0.45 µm membrane filter prior to analysis. A 10 µL aliquot of each filtrate was injected into the chromatographic system: six injections for test solutions and tree for reference solutions.

### Chromatographic System

The analysis of amoxicillin samples was performed using a high-performance liquid chromatography (HPLC) system.[14].^14^ The system consisted of an LC-2000 Plus Jasco (Jasco Corporation, Tokyo, Japan), equipped with a PU-2080 pump, an AS-2055 autosampler, a CO-2067 column oven, and a UV-2075 detector. Separation was performed using an Agilent Eclipse Plus C18 column (3.5 µm, 4.6 × 150 mm) with an isocratic mobile phase of diluent solution and acetonitrile (96:4, v/v) at a flow rate of 1.2 mL/min. The column temperature was maintained at 40 °C, and detection was carried out at 230 nm.

### Dissolution Test

This test was performed according to Mexican Pharmacopoeia specifications for capsules containing 500 mg of amoxicillin. The study was carried out using an Agilent 708-DS dissolution apparatus (Apparatus 2, paddle method; Agilent Technologies, Santa Clara, CA, USA). The acceptance criterion required that at least 80% of the labeled amoxicillin be dissolved within 60 min. The test was conducted using six capsules, and if this set did not comply, an additional six units were tested, in according with pharmacopoeial requirements [14].

### Infrared Analysis

Infrared (IR) spectra were obtained from capsule two brands sourced from the unregulated market (BN and CN), two from authorized pharmacies (BA and DA), and from the reference substance. For each brand, the contents of ten capsules were combined and homogenized. Approximately 10 mg of the powder mixture and 10 mg of reference standard, were placed directly onto the ATR crystal of a Spectrum Two N FT-NIR Spectrometer (PerkinElmer, Inc., USA) equipped with a diamond ATR accessory. Spectra were recorded as percent transmittance (%T) over the range of 4000–400 cm^−1^, using four scans per sample [14].

### Comparative In Vitro Dissolution Study

In vitro dissolution studies were conducted using 12 capsules per brand, in accordance with the Mexican Pharmacopoeia specifications for amoxicillin capsules [14]. The studies used an Agilent 708-DS apparatus (Apparatus 2) at 75 rpm. The dissolution medium consisted of 900 mL of degassed distilled water maintained at 37 ± 0.5 °C. Aliquots of 5 mL were manually withdrawn at 5, 10, 20, 30, and 60 min without volume replacement. Samples were filtered and appropriately diluted with distilled water before analysis. The dissolved amoxicillin was quantified using a UV–Vis spectrophotometer (Lambda XLS + , PerkinElmer, Inc., Shelton, CT, USA) at 272 nm, using a calibration curve with the reference standard. The regression equation from the validated method was used for the quantification [15].

The percentage of dissolved API was calculated based on the amount of amoxicillin in the collected samples and expressed as dissolution percentage curves over time. To compare dissolution profiles, the similarity factor (f₂) was calculated using brand DA as the reference product (designated as a national reference product as defined by the Mexican regulatory authority) [15, 16], according to the following equation.

$$ f_{2} = 50 \cdot \log \left\{ {\left[ {1 + \frac{1}{n}\sum\limits_{t = 1}^{n} {\left( {R_{t} - T_{t} } \right)^{2} } } \right]^{ - 0.5} \times 100} \right\} $$

where *n* is the number of sampling time points used to obtain the drug dissolution curve, *Rt* and *Tt* represent the cumulative percentages of the API dissolved from the reference product and the test product, respectively [15, 16].

### Dissolution Efficiency (DE)

Dissolution efficiency [17] was determined by calculating the area under the dissolution curve (AUC), which represents the percentage of API dissolved (*Q*) as a function of time, within the interval in which 100% of the labeled content was reached. The DE was calculated using the following equation:

$$ DE = \frac{{AUC_{0}^{t} }}{{Q_{100} t}} 100 \% $$

where *t* corresponds to time.

### Mean Dissolution Time (MDT)

The mean dissolution time was calculated from dissolution data to describe the rate and extent of drug release. This parameter was determined from the amount of API dissolved (*Q*) at each sampling point, relating the cumulative dissolution profile to time [17]. The MDT indicates drug release kinetics, with higher values suggesting a slower release rate.

$$ MDT = \frac{{\mathop \sum \nolimits_{0}^{\infty } \left( {ti\Delta Qt} \right)}}{{Q^{\infty } }} $$

where.

*ti* = average between *ti* and *ti—1.*

*t* = time.

*ΔQ* = amount of API dissolved between *ti* and *ti—1.*

*Q∞* = amount of API dissolved at infinite time.

### Statistical Analysis

Data are presented as the mean ± SD. Dissolution performance was evaluated using f₂, %DE, and MDT, with f₂ calculated against the reference product according to established regulatory criteria. Comparisons of %DE and MDT among the different brands were analyzed using ANOVA model including brand and point of sale as main factors, accounting for the unbalanced factorial structure of the design. When significant differences were detected (*p* < 0.05), Dunnett’s test was applied using brand DA as the reference.

## Results

### External Aspect

Visual inspection of the amoxicillin capsule brands revealed no appreciable differences in size, shape, or texture. All samples consisted of two-tone capsules, with subtle variations in the saturation of the green color (Table 2). Regarding primary packaging, all capsule brands were presented in blister packs, except for brand CN, which was packaged in an opaque white high-density polyethylene (HDPE) bottle.

**Table 2:** External appearance of the 500 mg amoxicillin hard gelatin capsules subjected to the study

Code	Size* (mm)	Form	Color	Aspect	Primary packaging
AN	20.00 ± 0.13	√	White/intense Green	√√	√√√
BN	20.23 ± 0.14	√	White/blue	√√	√√√
CN	19.88 ± 0.04	√	White/blue	√√	Opaque white high-density polyethylene bottle
DN	21.82 ± 0.08	√	White/soft Green	√√	√√√
AA	20.08 ± 0.02	√	White/intense Green	√√	√√√
BA	19.88 ± 0.12	√	White/blue	√√	√√√
DA	21.84 ± 0.10	√	White/soft Green	√√	√√√

^*^ Average value of n = 6 determinations ± standard deviation. √ = Cylindrical with semi-spherical bottom; √√ = Bicolor capsule with smooth, homogeneous texture without cracks; √√√ = Blister packs

**Fig. 1:**
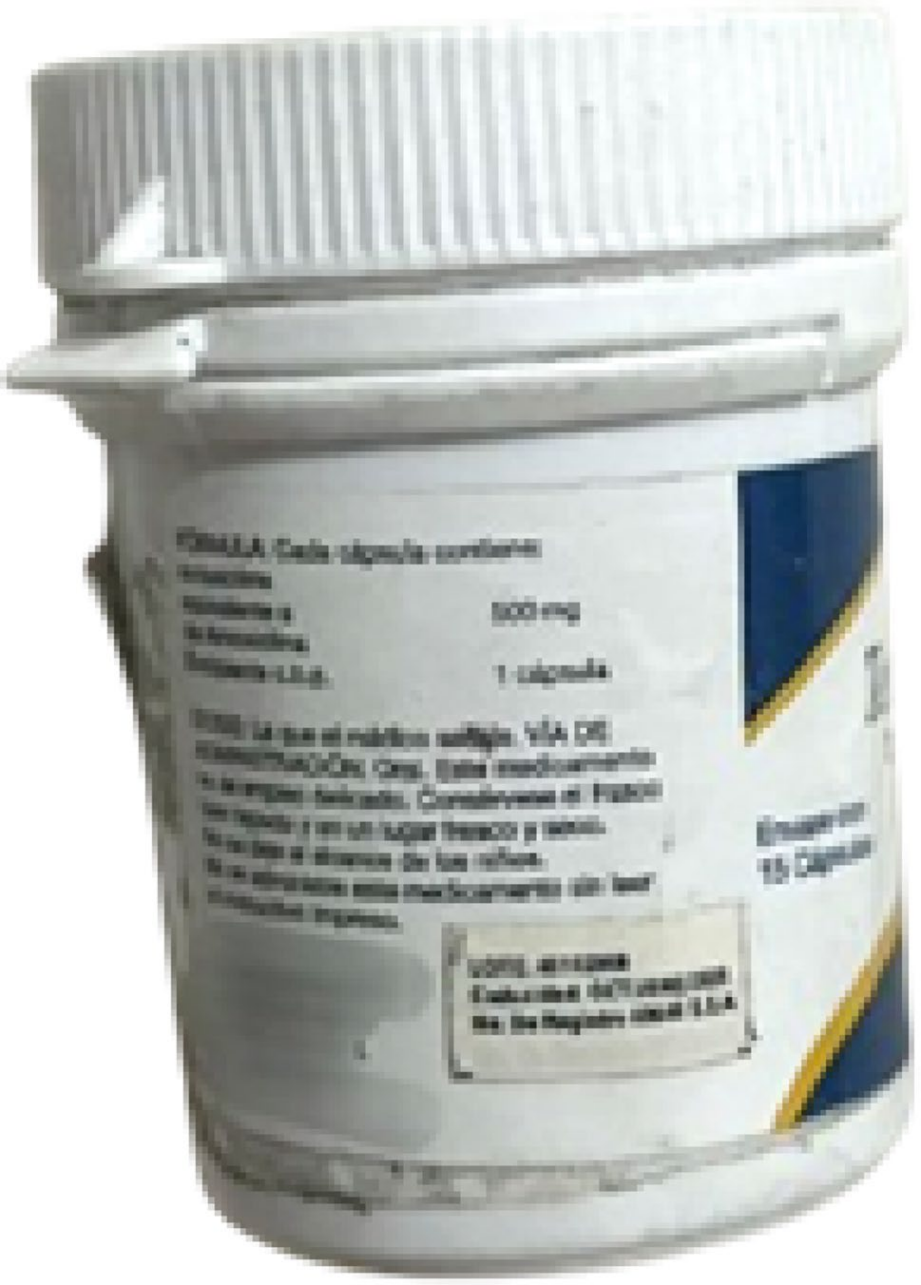
Primary packaging of the CN brand. The package displays an overlay label containing the expiration date and lot number information

During inspection of the CN bottle, a rectangular overlay label was observed, displaying an expiration date of *October 2026* and lot number *45102008* (Fig. 1). Upon removal of the overlay, a different expiration date—*October 2022*—was revealed, printed directly on the container surface.

The results of the quality control tests conducted on all brands of 500 mg amoxicillin hard gelatin capsules are summarized in Table 3. At least one deviation from the established pharmacopoeial specifications was identified in all products obtained from the unregulated market.

**Table 3:** Quality control test results for 500 mg amoxicillin hard gelatin capsules

Control test	Brands						
	DA	DN	BA	BN	AA	AN	CN
Assay ^*^(%)	112.39 ± 4.39	120.48 ± 4.55	111.54 ± 2.99	111.90 ± 1.54	107.53 ± 3.74	115.08 ± 3.10	81.68^###^ ± 6.64
Weigh variation^**^ (mg)	608.90 ± 9.59 (595.3—620.5)	589.81 ± 9.90 (574.8—607.6)	622.81 ± 16.98(576.7—632.7)	613.38 ± 18.50 (587.7—639)	582.86 ± 3.53 (574—588.2)	582.30 ± 4.95 (577.7—587.6)	558.22 ± 24.89 (519.9—604.4)
Dose uniformity L1 value^***^	7.576	22.886^###^	9.951	7.272	2.035	1.682	16.208^###^
Desintegration (min)^#^	2.32	1.45	2.35	3.04	1.41	2.3	2.07
%Q 60 min^##^	80.71 ± 6.33	87.945 ± 2.39	80.583 ± 5.10	68.082 ± 8.96^###^	82.681 ± 15.21	73.989 ± 577^###^	68.253 ± 6.01^###^

^*^Assay: Not less than 90.0% and not more than 120.0% of the amoxicillin content.

**Data presentation: Mean of *n* = 10, with minimum and maximum values reported. ^***^ Dose uniformity: L1 < 15%. ^#^ Disintegration time: Not more than 15 min. ^##^ Dissolution (12 units): Average of 12 units ≥ 80%. ^###^ A complete pharmacopoeial evaluation is required to confirm

In the spot dissolution test, the first acceptance criterion (Stage 1, S₁), which requires that for six units, each individual value of *Q* is not less than *Q* + *5,* [14] was not met in any tested batches of amoxicillin capsule brands. After application of the second acceptance criterion (Stage 2, S_2_), which involves dissolution testing of an additional six units, the batches of brands purchased from regulated retail pharmacies complied with the specification, meeting the requirement that the average of 12 units is equal to or greater than Q, and that no individual unit is less than Q—15%. In contrast, the brands acquired from the unregulated market showed unsatisfactory dissolution performance in the evaluated stages, although the full pharmacopoeial sequential test could not be completed. Among them, brand CN exhibited the greatest number of deviations from pharmacopoeial requirements. The percentage of API determined in the assay was 81.68 ± 6.64%, which is below the specified range of 90.0–120.0%. The dose uniformity test (Table 3), performed on 10 units per brand, showed variability in amoxicillin content among products. Most brands met the pharmacopoeial limit (L1 ≤ 15), [14] but two brands (CN and DN) exceeded it (16.208 and 22.886), indicating greater variability between units. Further evaluation (L2/L3) [14] was not possible due to limited samples, so compliance with pharmacopoeial requirements could not be definitively established.

### Infrared Spectral Analysis

**Fig. 2:**
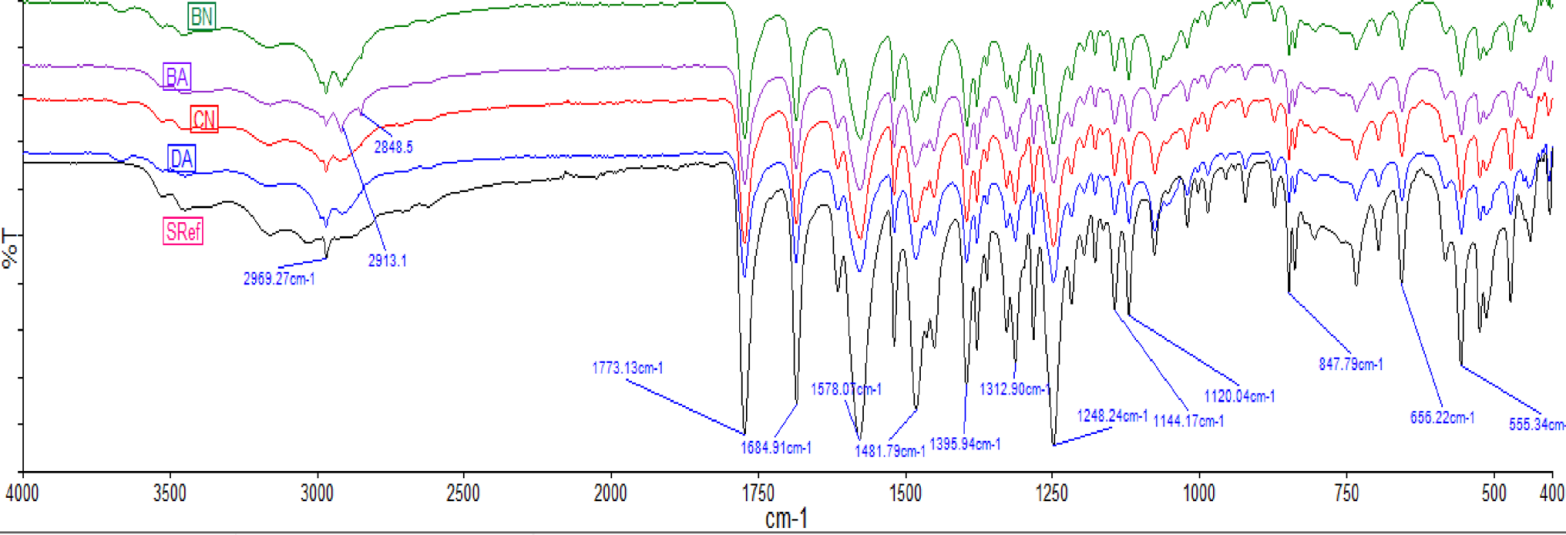
FTIR spectrum of samples BN, BA, CN, DA and Reference substance (4000 to 400 cm^−1^), exhibiting slight variations below 3000 cm^−1^ and marker similarity between 2000 to 400 cm^−1^

**Fig. 3:**
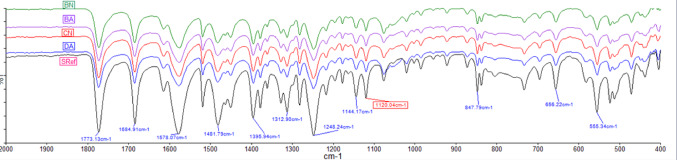
FTIR spectra of samples BN, BA, CN, DA, and the reference substance in the 2000–400 cm^−1^ range, showing significant similarity among all analyzed signals

Four of the seven capsule brands were analyzed by spectroscopy FTIR, the IR spectra of the samples BN, CN, BA, DA, and reference standard (SRef) of amoxicillin are shown in Figs. 2 and 3.

Comparison with the SRef spectrum revealed that in the 3500–2500 cm⁻^1^ region, the four analyzed samples exhibited minor differences in signals corresponding to CH₂ and CH₃ groups (hydrocarbon moieties). For the BA brand, bands at 2913.1 and 2848.5 cm^−1^ were observed with greater intensity. However, in the 2000–400 cm^−1^ fingerprint region, no significant changes were observed in either the position or shape of the signals, indicating that the chemical structures of amoxicillin were largely preserved (Fig. 3).

### Dissolution Study

**Fig. 4:**
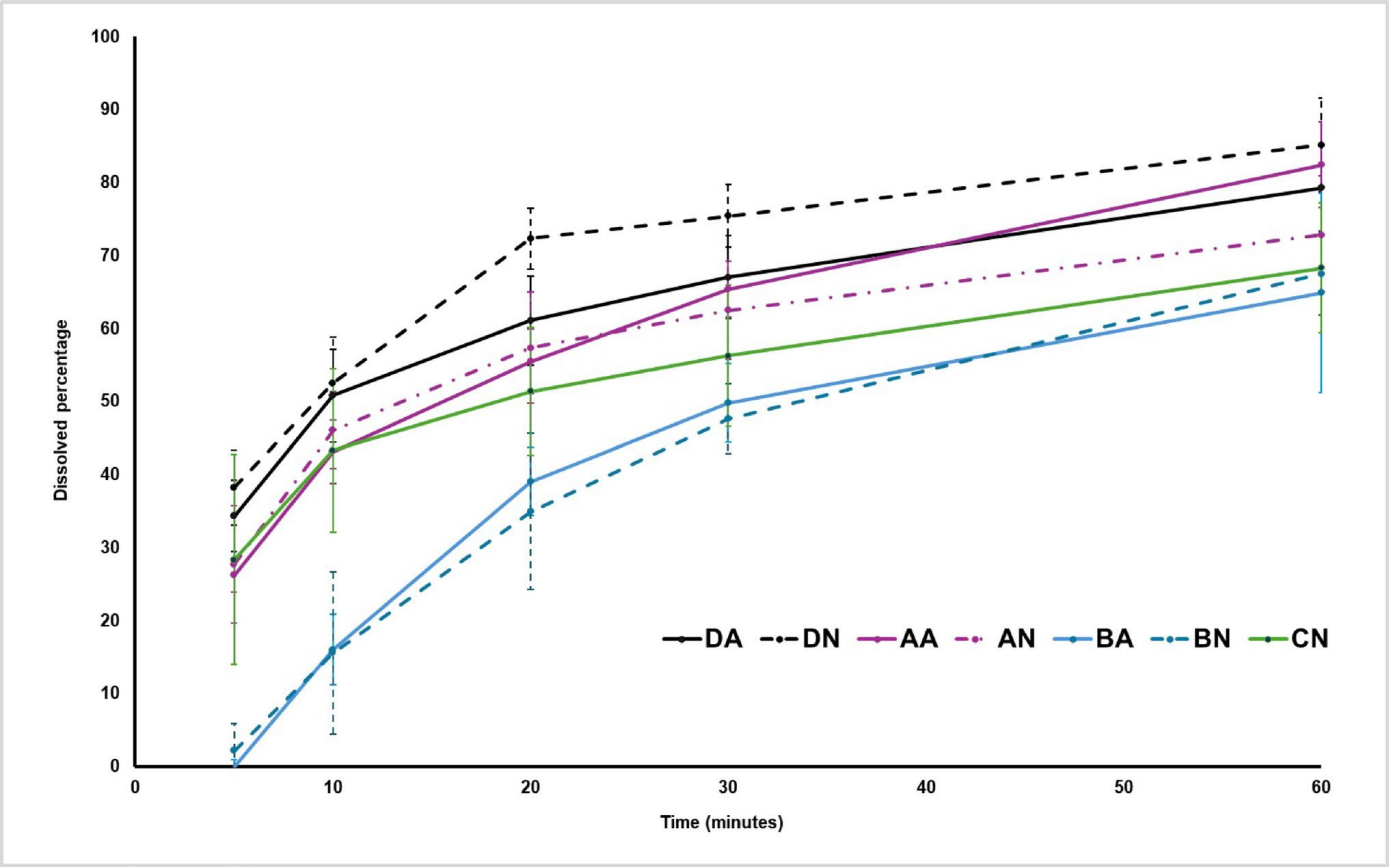
Average dissolution profiles of amoxicillin capsules purchased from formal and informal points of sale. Each point represents the mean of 12 determinations ± standard deviation

The comparative behavior of the dissolved percentage of amoxicillin contained in the capsules over the 5- to 60-min interval for the seven different brands studied is shown in Fig. 4. None of the brands reached an average dissolved percentage of amoxicillin above 90%. Brand BA and BN, regardless of where they were purchased, authorized pharmacies or an unregulated market known as Tepito tianguis, presented the lowest percentage of amoxicillin dissolved in the first 20 min (between 35 and 40%), compared to the rest of the brands. In contrast, reference brands DA and DN presented the highest percentage of dissolved amoxicillin at 20 min (between 60 and 70% of dissolved amoxicillin).

The similarity criterion for dissolution profiles between amoxicillin brands purchased from authorized pharmacies and the unregulated market, with respect to the reference brand (DA), was established as *f₂* ≥ 50. This criterion was met by all brands except BA and BN, whose *f₂* values were below the stipulated threshold, as shown in Table 4.

**Table 4:** Values of *f₂,* dissolution efficiency (%DE), and mean dissolution time (MDT) obtained for amoxicillin capsule brands

Brand	f₂	DE (%)	MDT* (min)
AN	63.23	56.65 ± 6.46	9.69 ± 1.67
BN	28.95	40.76 ± 4.16**	16.04 ± 4.06**
CN	51.71	52.17 ± 4.15**	9.68 ± 1.37
DN	57.32	68.23 ± 4.01**	10.15 ± 2.03
AA	62.50	59.23 ± 3.61	13.92 ± 1.72
BA	39.88	41.36 ± 5.31**	15.43 ± 3.40**
DA	–	61.54 ± 5.03	10.63 ± 1.92

^*^Data represented as mean ± standard deviation of twelve determinations

^**^Statistically significant difference (*p* < 0.05) compared with the reference brand (DA). *f₂* was calculated using the DA brand as the reference

The ANOVA revealed significant differences among brands for %ED (F(6,77) = 55.702, *p* < 0.001) and TMD (F(6,77) = 16.084, *p* < 0.001), using Dunnett´s test identified significant indicated inter-brand differences relative to the reference brand (DA).

Dissolution efficiency was generally lower for the brands acquired in the unregulated market compared with the DA brand. Brands BN and CN purchased from the unregulated market exhibited the lowest efficiency in releasing the drug from the dosage form. This finding suggests reduced amoxicillin availability for systemic absorption and, consequently, a diminished therapeutic effect. These brands also showed longer mean dissolution times, as presented in Table 4.

## Discussion

Purchasing medicines from unauthorized retailers poses a potential health risk due to possible exposure to SMP or FMP. This study demonstrates that samples of amoxicillin capsules purchased from an unregulated market in Mexico City (Tepito tianguis) exhibited marked irregularities in their quality attributes, particularly in terms of API content, dosage uniformity, and dissolution behavior. Consequently, these medicines could threaten patient health by failing to ensure safety and therapeutic efficacy. The findings are consistent previous evidence from Mexico and the border region indicating variations in pharmaceutical performance among multi-source products [11, 18–20]. For example, a study comparing products obtained in Mexico and the United States, reported differences in quality attributes, such as content uniformity and weight variation when analyzing different brands and points of sale [19]. Regarding regulated versus unregulated markets, international evidence indicates that the proportion of SMP increases when procurement occurs outside the regulated supply chain, although the magnitude varies by country and API evaluated. In Haiti, a field study in street markets found that more than half of the samples exhibited poor spectral agreement with their reference standards for several antimicrobials (especially macrolides and quinolones), although amoxicillin showed high agreement in most evaluated units [21].

Amoxicillin 500 mg capsules were selected for this study because this antibiotic is among the most frequently reported as falsified medical product worldwide [10, 18]. The samples were obtained from the largest unregulated street market in Mexico City, known as the Tepito tianguis, located in the northern part of the historic center and is characterized by its intense commercial activity [6]. The decision to sample a single unregulated market was deliberate and aligned with the exploratory and comparative design of the study. The objective was not to estimate city-wide prevalence, but to compare pharmaceutical quality between regulated and unregulated distribution points of sale under controlled conditions. Expanding sampling to multiple informal markets would likely have introduced additional heterogeneity in sourcing and storage practices, potentially confounding the interpretation of regulatory status as a fixed study factor. During the purchasing visits, several brands of 500 mg amoxicillin were available; however, only four brands were obtained in sufficient quantities and from the same batch to perform the tests proposed in this study. All medicines acquired from this market were unsecured and directly exposed to environmental conditions.

The comparison group consisted of commercial amoxicillin capsule brands purchased from authorized pharmacies, where storage conditions were appropriately controlled. One of the four brands corresponded to the reference product, defined as that designated by the regulatory authority, with a valid health registration and commercially available [15]. The remaining brands were generic products.

As shown in Table 1, the product identified as “CA” was excluded because it was not available in regulated retail pharmacies within the metropolitan area of Mexico City. Although the CN product displayed a health registration number on its packaging, verification with COFEPRIS [22] confirmed that the corresponding registration had been cancelled. Therefore, this product should be considered to lack valid marketing authorisation. This regulatory status may explain why it was not possible to find in regulated pharmacies. Additionally, the presence of an altered expiration date label further supports the classification of this sample as a falsified [2] or, at minimum, an unregistered medical product. Despite containing amoxicillin, such discrepancies raise significant concerns regarding its quality, safety, and traceability. This observation is consistent with global reports estimating that approximately 5% of antibiotics marketed worldwide are falsified medical products [23, 24].

According to the WHO, substandard medicines are authorized medical products that fail to meet quality standards, whereas falsified medicines are those deliberately and fraudulently mislabeled with respect to their identity or origin [2]. Failure to comply with quality specifications may result from inadequate storage, manufacturing, or distribution practices, or from products being sold beyond their expiration dates. [23]

All amoxicillin capsule samples analyzed in this study exhibited variations in color (Table 2), ranging from blue (BN, BA, and CN) to green (DA, DN), with some showing greenish hues (AA and AN). However, no color differences were observed when comparing each product with its corresponding unregulated counterpart, suggesting that the products within each brand might share the same manufacturing origin. Expiration dates ranged from September 2024 (DA) to October 2026 (CN).

Differences in the physical characteristics of pharmaceutical equivalents, such as tablet or capsule size and shape, may influence therapeutic adherence and patient acceptability. Oversized oral solid dosage forms (> 22 mm) can cause swallowing difficulties and are associated with risks such as esophagitis, ulcers, or delayed transit time. [12] This concern did not apply to the amoxicillin capsules analyzed in this study, regardless of the point of purchase. As Table 2 indicates, the DA and DN were the largest (21.82 ± 0.08 mm and 21.84 ± 0.10 mm, respectively) compared to other brands (approximately 20 mm). However, none exceeded the 22 mm limit.

Disintegration time and drug dissolution percentages among pharmaceutical equivalents are often influenced by dosage form size. Larger solid oral medications may display different disintegration and dissolution behaviors due to increased surface area contact with the dissolution medium [25]. Nevertheless, in this study, no direct correlation was observed between capsule size and disintegration time or the percentage of amoxicillin dissolved at 60 min (%Q₆₀min) (Tables 2 and 3). These results suggest that other factors such as formulation characteristics, storage conditions, or packaging type of the medicines purchased in the unregulated market may have a greater influence on the dissolution performance than capsule size itself [24, 26]. This suggestion is reinforced by the observation that all analyzed brands purchased from regulated establishments met the official dissolution specification at Stage 2 (S2). While brands purchased from unregulated markets (BN, AN, and CN) exhibited poor dissolution performance and marked variability among individual units, the pharmacopoeial sequential dissolution acceptance procedure could not be fully completed because an insufficient number of capsules was available to perform Stage 3 (S3) [14]. Therefore, from strict pharmacopoeial perspective, the dissolution evaluation for these products should be considered incomplete. Nevertheless, the results obtained at S1 and S2 already demonstrated substantial variability and a clear tendency toward inadequate dissolution behavior, which are relevant indicators of inconsistent product quality [27]. This limitation has been acknowledged, and the findings should be interpreted with appropriate caution. Regardless of the place of purchase, all medications examined declared a concentration of 500 mg of amoxicillin on their labels. According to the results obtained from the assay test (Table 3), five of the seven analyzed medicines met the established specification (drug content between 90 and 120% of the labeled amount) [14]. However, the DN and CN brands did not meet the pharmacopoeial specification, [14] as their amoxicillin content was either excessive (DN = 120.48%) or insufficient (CN = 81.68%). This notable variability clearly indicates deficiencies in product quality [24].

To ensure therapeutic benefit, dosage units such as tablets, capsules, or other solid single-dose preparations must contain an amount of active ingredient that falls within a narrow range of the concentration declared on the label. For this purpose, the pharmacopoeia defines two control methods: the dose uniformity test and a simplified alternative, the mass variation test [27, 28]. The results of the dose uniformity test (Table 3), showed that although most brands complied with the compendial acceptance value (L1), the brands CN and DN exceeded the permitted limit, suggesting increases variability in the distribution of amoxicillin content among induvial capsules. According to the compendial method, these results require further sampling for a definitive compliance decision; however, this was not possible due to the limited number of units available for analysis. Therefore, these findings should be interpreted with caution and not as definitive evidence of non-compliance, but rather as indicative of potential inconsistencies in dosage unit uniformity. Variability in the content of antibiotic dosage forms is of particular concern, because may result in inconsistent exposure to the API, affecting its bioavailability and compromising the therapeutic efficacy of amoxicillin [8, 18, 21].

In this context, the variability observed in some of the evaluated brands may reflect deficiencies in manufacturing, quality control or distribution conditions. These results underscore the need to reinforce regulatory oversight and post-market surveillance, especially for medicines from unregulated channels, whether physical or online [4]. Additional studies with full pharmcopoeial evaluation are necessary to confirm these findings.

Furthermore, analysis of the infrared (IR) spectra for samples BN, CN, BA, and DA revealed similarity with the reference amoxicillin spectrum in the region between 2000 and 400 cm^−1^, which includes the fingerprint region. The correspondence in the position and shape of the absorption bands indicates that the API was present without chemical alteration in all medical products analyzed. Minor spectral differences were observed below 3000 cm^−1^ in the bands assigned to CH₂ and CH₃ groups, which may be attributed to the presence of formulation excipients. Bands within the 3000—2850 cm^−1^ region are characteristic of long aliphatic chains and are reported in lipophilic materials including pharmaceutical excipients such as magnesium stearate [29]. The increased intensity of the bands at 2913.1 and 2848.5 cm^−1^ in the powder mixture from brand BA, compared with the reference amoxicillin, may therefore reflect the spectral contribution of aliphatic excipients present in the formulation. This effect could also have been favored using ATR with a diamond crystal, a technique highly sensitive to surface composition that can highlight hydrophobic components exhibiting better contact with the crystal [30]. These variations, however, do not affect the molecular structure of the API. Since the degradation-sensitive regions of amoxicillin (fingerprint region 1200—990 cm^−1^) show significant changes, the spectra obtained do not indicate global structural modifications (Fig. 3).

Given the differences observed in pharmacopoeial quality tests, regardless of the source of acquisition, and considering that in vitro dissolution behavior can be predictive of in vivo performance, such as bioavailability [31]. Dissolution profiles were obtained for all products analyzed. The comparison of mean dissolution profiles (Fig. 4) revealed a slower dissolution process over the 0-to-60-min interval, particularly in generic medical products purchased from unregulated markets (AN, BN, CN), except for the reference pharmaceutical product acquired from the same source (DN).

Of particular concern was the poor dissolution performance of the BA and BN products, whose dissolution profiles were markedly slower than those of the other formulations. This was reflected in their lower dissolution efficiency (%DE) and longer mean dissolution times (MDT), as shown in Table 4.

The ANOVA confirmed statistically significant differences among brands for both %DE and MDT, indicating that the observed differences in API release performance were not attributable to random variability. Dunnett´s test confirmed significant differences relative to the reference product (DA), supporting the presence of measurable inter-brand variability in dissolution performance.

In the case of medicines obtained from unregulated market, the low dissolution percentages within the first 60 min and high variability observed were expected outcomes, given their likely exposure to inadequate storage and distribution conditions factors that negatively affect API release and, consequently, its bioavailability.

Notably, even among products purchased from authorized pharmacies (both reference and generic), no consistent similarity was observed in their dissolution profiles, contrary to what would be expected under regulated manufacturing and distribution conditions. Only the reference products (DA and DN) showed comparable dissolution behavior. This inconsistency raises questions about the pharmaceutical equivalence of certain medical products, particularly those of brand B, suggesting that the differences observed in this amoxicillin formulation may stem from variations in formulation or manufacturing processes rather than from the point of sale.

## Conclusion

Amoxicillin capsule samples obtained from unregulated retail outlets in Mexico City showed greater variability in key quality attributes than those from regulated establishments. Despite the limited sample availability, which prevented a complete pharmacopoeial assessment for some tests, variability found in assay, uniformity of the dosage unit (L1), and the dissolution suggest inconsistent pharmaceutical quality. Although definitive non-compliance could not be established for all samples, the findings raise concerns regarding product consistency and the potential risks associated with antibiotics from unregulated markets.

### Study Limitations

The study face limitations due to the small number of amoxicillin capsule brands from unregulated markets, difficulties in obtaining sufficient batch units, and restricted procurement limited to a physical market in Mexico. As a result, some pharmacopoeial test could not be completed, findings should be interpreted cautiously, and results may not represent all unregulated markets, though they highlight variability in pharmaceutical quality and the need for further research.

## Data Availability

All data supporting the findings of this study are available within the paper.
